# Urinary catecholamine excretion, cardiovascular variability, and outcomes in tetanus

**DOI:** 10.1186/s41182-023-00512-0

**Published:** 2023-03-30

**Authors:** Duc Hong Du, Nguyen Quan Nhu Hao, Nguyen Van Hao, Tran Tan Thanh, Huynh Thi Loan, Lam Minh Yen, Tran Thi Diem Thuy, Duong Bich Thuy, Nguyen Thanh Nguyen, Nguyen Thi Phuong Dung, Evelyne Kestelyn, Ha Thi Hai Duong, Nguyen Thanh Phong, Pham Thi Tuyen, Nguyen Hoan Phu, Ho Dang Trung Nghia, Bui Thi Bich Hanh, Pham Kieu Nguyet Oanh, Phan Vinh Tho, Phung Tran Huy Nhat, Phan Nguyen Quoc Khanh, Duncan Wyncoll, Nicholas P. J. Day, Nguyen Van Vinh Chau, H. Rogier van Doorn, Le Van Tan, Ronald B. Geskus, C. Louise Thwaites

**Affiliations:** 1 Oxford University Clinical Research Unit, Ho Chi Minh City, Vietnam; 2 University Medical Center, Ho Chi Minh City, Vietnam; 3 Hospital for Tropical Diseases, Ho Chi Minh City, Vietnam; 4 Centre for Tropical Medicine and Global Health, University of Oxford, Oxford, UK; 5 Pham Ngoc, Thach Medicine University, Ho Chi Minh City, Vietnam; 6 Guys and St, Thomas’ Hospitals, London, UK; 7 Mahidol Oxford Research Unit, Bangkok, Thailand

**Keywords:** Catecholamine, Tetanus, Infectious diseases, Cardiovascular, Mechanical ventilation, Autonomic nervous system dysfunction, Intensive care

## Abstract

Severe tetanus is characterized by muscle spasm and cardiovascular system disturbance. The pathophysiology of muscle spasm is relatively well understood and involves inhibition of central inhibitory synapses by tetanus toxin. That of cardiovascular disturbance is less clear, but is believed to relate to disinhibition of the autonomic nervous system. The clinical syndrome of autonomic nervous system dysfunction (ANSD) seen in severe tetanus is characterized principally by changes in heart rate and blood pressure which have been linked to increased circulating catecholamines. Previous studies have described varying relationships between catecholamines and signs of ANSD in tetanus, but are limited by confounders and assays used. In this study, we aimed to perform detailed characterization of the relationship between catecholamines (adrenaline and noradrenaline), cardiovascular parameters (heart rate and blood pressure) and clinical outcomes (ANSD, mechanical ventilation required, and length of intensive care unit stay) in adults with tetanus, as well as examine whether intrathecal antitoxin administration affected subsequent catecholamine excretion. Noradrenaline and adrenaline were measured by ELISA from 24-h urine collections taken on day 5 of hospitalization in 272 patients enrolled in a 2 × 2 factorial-blinded randomized controlled trial in a Vietnamese hospital. Catecholamine results measured from 263 patients were available for analysis. After adjustment for potential confounders (i.e., age, sex, intervention treatment, and medications), there were indications of non-linear relationships between urinary catecholamines and heart rate. Adrenaline and noradrenaline were associated with subsequent development of ANSD, and length of ICU stay.

**Supplementary Information:**

The online version contains supplementary material available at 10.1186/s41182-023-00512-0.

## Introduction

Tetanus is a severe disease caused by tetanus toxin, a powerful neurotoxin released by *Clostridium tetani.* Tetanus toxin inhibits central inhibitory neuronal synapses, resulting in motor neuronal disinhibition and characteristic muscle spasm [1]. A similar action on the autonomic nervous system is believed to cause the cardiovascular manifestations of severe tetanus. Historical studies report elevated adrenaline, noradrenaline or their metabolites in urine of patients with autonomic nervous system dysfunction (ANSD) but studies failed to correct for confounders [2–5]. Rapidly fluctuating blood pressure remains one of the most difficult aspects of tetanus management. A better understanding of the relationship between autonomic nervous system activity, catecholamines and cardiovascular parameters, including blood pressure would aid decision-making and therapeutic intervention.

The objective of the study was to examine the relationship between urinary catecholamines (adrenaline and noradrenaline), cardiovascular parameters and disease outcomes using data from patients enrolled in a randomized clinical trial investigating the role of intrathecal antitoxin in tetanus treatment [6].

## Methods

The study was carried out at the Hospital for Tropical Diseases (HTD), a 700-bed infectious diseases hospital in southern Vietnam.

Patients included in this study were selected from those enrolled in a factorial randomized controlled trial where the intervention consisted of randomization to either intrathecal human tetanus immunoglobulin or sham procedure and to either human or equine intramuscular antitoxin (both interventions given on admission). Full entry criteria for the trial are given in the supplement, but included adult patients with a clinical diagnosis of generalized tetanus and excluding those already receiving mechanical ventilation (MV) or expected to require this imminently.

For this study, we included all patients enrolled in the randomized controlled from whom a 24-h urine collection had been obtained on the 5th day of hospitalization. Patients receiving vasopressors 24 h before or during urinary collections were excluded. All patients were treated according to standard hospital protocol using benzodiazepines for spasm control, adding magnesium sulphate or pipecuronium/MV as clinically indicated [7]. Patients were followed daily by study staff who recorded clinical parameters including maximum/minimum heart rate, blood pressure, daily drug use, events of MV and ANSD. ANSD was diagnosed clinically and defined as the presence of at least three of the following signs occurring within 12 h of each other: heart rate > 100 bpm, systolic blood pressure > 140 mmHg, blood pressure fluctuation with minimum mean arterial pressure < 60 mmHg, and temperature > 38 °C without other clinically apparent cause. Catecholamines (adrenaline and noradrenaline) were measured from 24-h collections taken into hydrochloric acid (pH < 3) on day 5 of hospitalization using enzyme-linked immunosorbent assay (ELISA). Day 5 was selected as the day ANSD is characteristically identified (median 5 days after hospital admission) [8].

The study was approved by the Ethics Committee of HTD, Oxford Tropical Research Ethics Committee and the Vietnamese Ministry of Health. All patients gave written informed consent.

Numerical variables are reported as mean (standard deviation) or median [1^st^–3rd quartile (Q1–Q3)] depending on their distribution. Categorical variables are presented as total number with percentages. To assess the association between catecholamines in 24-h collected urine and cardiovascular outcomes, linear regression models were fitted. We used restricted cubic splines with three knots (the knots were chosen at the 10%, 50% and 90% percentile of variable values) to allow for potential non-linear relationships. We performed both unadjusted analyses and analyses adjusted for age, sex, study interventions (intrathecal and intramuscular treatment), doses of benzodiazepines and pipecuronium indicated during first 5 days of treatment. We used the Box–Cox procedure to find a suitable transformation of the outcome variable. We conducted both unadjusted and multivariable logistic regression models to investigate the association between catecholamines (linear and non-linear trends) and MV and ANSD, occurring after the 5^th^ day. We used linear regression to examine the association between catecholamines and the length of intensive care unit (ICU) stay, using the Box–Cox procedure to find a suitable transformation. Multivariable models for cardiovascular outcomes, binary outcomes and ICU stays were adjusted for potential confounding factors measured at baseline or during the first 5 days of hospitalization (age, sex, intrathecal and intramuscular interventions, and medications indicated). All statistical analyses were carried out using R (version 4.0.4).

## Results

272 patients were enrolled in the randomized trial between January 2017 and September 2019. Of these, three died before day 5 of hospitalization, leaving 269 with 24-h urine collections eligible for inclusion in this study. Six of these patients received intravenous vasopressors or inotropes (adrenaline, noradrenaline) and were excluded, leaving samples from 263 patients for analysis. Baseline demographics, clinical data and outcomes are shown in Table 1.

**Table 1:** Descriptions of the demographic and clinical features of tetanus patients

Characteristic	*N*	Median [Q1–Q3], mean (SD) or count (%)
Age (years)	263	49 (14)
Male sex	263	222 (84.4%)
BMI (kg/m^2^)	263	21.33 [19.82, 23.16]
Coexisting comorbidities	263	121 (46.0%)
Duration of illness (days)	263	3 [2, 5]
Incubation period (days)	202	9 [6, 14]
Period of onset (h)	230	48 [24, 72]
Ablett Score* on admission	263	
I		47 (17.9%)
II		196 (74.5%)
III		20 (7.6%)
APACHE II Score	263	3 [2, 7]
SOFA Score	263	
0		228 (86.7%)
≥ 1		35 (13.3%)
Tetanus Severity Score**	263	0 [− 3, 4]
Outcome	263	
In-hospital death or palliative discharge		2 (0.8%)
Transfer to other facility		6 (2.3%)
Alive hospital discharge		255 (97.0%)
ICU length of stay (days)	261	14 [8, 22]
Mechanical ventilation during hospitalization	263	123 (46.8%)
Autonomic nervous system disturbance (ANSD)	263	49 (18.6%)

*Ablett score [8]: Grade I mild tetanus, no spasm; Grade II presence of muscle spasms but not interfering with breathing; Grade III severe muscle spasms interfering with breathing

**Tetanus Severity Score: a composite score of 9 domains of variables on admission with higher scores associated with worse outcome. Scores of > 7 indicate high likelihood of poor outcome [9]

Results of catecholamine analysis showed median [Q1–Q3] value of adrenaline 175 [83, 428] nmol/day and noradrenaline 645 [321, 1392] nmol/day. There was no evidence of an effect of the trial treatment intervention (Additional file 1: Table S6).

**Fig. 1.**
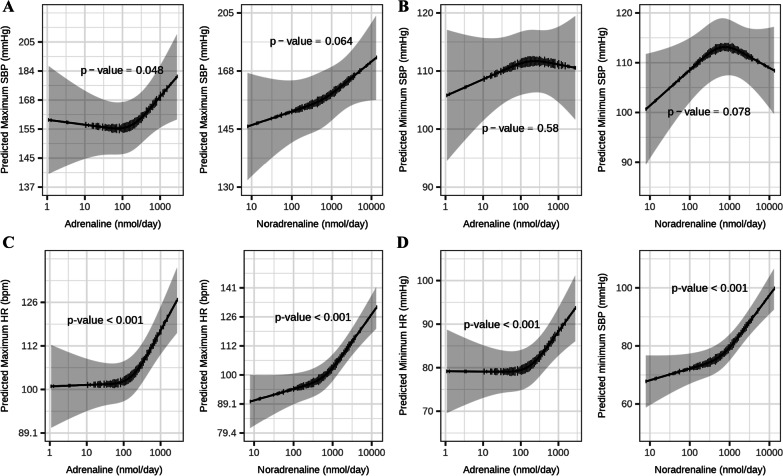
24-h urinary catecholamine excretion (nmol/day) and cardiovascular parameters on the 5th day of treatment. **A** Maximum systolic blood pressure (SBP), **B** minimum systolic blood pressure, **C** maximum heart rate (HR), and **D** minimum heart rate; *mmHg* millimeter of mercury, *bpm* beats per minute. The vertical lines represent the frequency counts of outcome variables. *P*-values indicate evidence for overall relationships between catecholamines and cardiovascular parameters measured from models via restricted cubic splines. All predictions are shown for age 48 years, male sex, equine intramuscular treatment interventions, and total doses of benzodiazepines of 2300 mg/day

Strong evidence of non-linear relationships were seen between urinary catecholamines and maximum and minimum heart rate. Weaker evidence of relationships between catecholamines and maximum and minimum systolic blood pressure were apparent (Fig. 1).

Examining the relationships with clinical outcomes, adrenaline and noradrenaline were associated with subsequent clinical diagnosis of ANSD during hospitalization (Table 2, Additional file 1: Table S2.1, S2.2, Fig. S2.1). Increased adrenaline from 100 nmol/day to 1000 nmol/day was associated with increased odds of developing ANSD (adjusted odds ratio (OR) (95% confidence intervals (CIs)): 8.9 (2.4–37.6), *p* = 0.002). Similarly, increased noradrenaline from 100 nmol/day to 1000 nmol/day was associated with increased odds of developing ANSD (adjusted OR (95% CIs): 11.4 (2.9–52.5), *p* < 0.001) (Table 2 and Additional file 1: Fig. S2.1). Similar relationships were indicated when allowing for non-linear trends (Additional file 1: Fig. S2.2).

**Table 2:** 24-h urinary catecholamine excretion on the 5th day of treatment and autonomic nervous system dysfunction (ANSD) after exclusion of patients developed ANSD before day 5 (*n* = 240)

Catecholamines	Median [Q1, Q3]		Multivariate model		
	No ANSD, *N* = 214 (89%)	ANSD, *N* = 26 (11%)	OR^1^	95% CI^1^	*p*-value
Log10 (adrenaline (nmol/day))	2.18 [1.85, 2.50]	2.75 [2.50, 2.92]	8.86	2.44, 37.60	0.002
Log10 (noradrenaline (nmol/day))	2.71 [2.45, 3.06]	3.17 [2.98, 3.40]	11.43	2.90, 52.45	< 0.001

^1^*OR* odds ratio per one unit increased on a log base 10 scale of the urinary catecholamine excretion measured (nmol/day), *CI* confidence Interval

Multivariate analyses: adjusted for age, sex, intrathecal and intramuscular treatment interventions, total dose of medications (benzodiazepines, pipecuronium) during the first 5 days

There was moderate-to-weak evidence that adrenaline and noradrenaline were associated with increased odds of subsequent MV (Table 3, Additional file 1: Table S3.1, S3.2, Fig. S3.1). There was also weak indication that catecholamines were associated with probability of subsequent MV during hospitalization if catecholamines were modeled nonlinearly via restricted cubic splines in adjusted models (Additional file 1: Fig. S3.2).

**Table 3:** 24-h urinary catecholamine excretion on the 5th day of treatment and mechanical ventilation required (MV) required after exclusion of patients required MV before day 5 (n = 157)

Catecholamines	Median [Q1, Q3]		Multivariate model		
	No MV, *N* = 140 (89%)	MV, *N* = 17 (11%)	OR^1^	95% CI^1^	*p*-value
Log10 (adrenaline (nmol/day))	2.04 [1.76, 2.51]	2.33 [2.12, 2.51]	3.00	0.70, 14.09	0.149
Log10 (noradrenaline (nmol/day))	2.59 [2.33, 2.84]	3.02 [2.73, 3.14]	4.23	0.94, 22.81	0.074

^1^*OR* odds ratio per one unit increased on a log base 10 scale of the urinary catecholamine excretion measured (nmol/day), CI = confidence Interval

Multivariate analyses: adjusted for age, sex, intrathecal and intramuscular treatment interventions, and total dose of benzodiazepines during the first 5 days

There was a strong positive linear association between adrenaline and length of ICU stay (Table 4 and Additional file 1: Fig. S4.1). Increased adrenaline from 100 nmol/day to 1000 nmol/day was associated with prolonged ICU stay (days) after adjusting for covariates (β (95% CIs): 11.2 (10.5–13.2), *p* = 0.005). Similarly, increased noradrenaline from 100 nmol/day to 1000 nmol/day was associated with prolonged ICU stay (days) after adjusting for covariates (β (95% CIs): 12.6 (11.0–14.1), *p* < 0.001). Similar relationships were indicated when allowing for non-linear trends (Additional file 1: Fig. S4.2).

**Table 4:** 24-h urinary catecholamine excretion on the 5^th^ day of treatment and length of ICU stay (days)

Catecholamines	Univariate			Multivariate		
	Beta^1^	95% CI^1^	*p*-value	Beta^1^	95% CI^1^	*p*-value
Log10 (adrenaline (nmol/day))	17.0	15.1–19.5	< 0.001	11.2	10.5–13.2	0.005
Log10 (noradrenaline (nmol/day))	20.4	17.8–22.9	< 0.001	12.6	11.0–14.1	< 0.001

^1^Beta = increase in length of ICU stay (days) with one unit increase of the urinary catecholamine excretion measured (nmol/day) on a log base 10 scale, *CI* confidence interval

Multivariate analyses: adjusted for age, sex, intrathecal and intramuscular treatment interventions, and total dose of medications (benzodiazepines, pipecuronium) during the first 5 days

## Discussion

Our paper describes urine catecholamine concentrations, measured using a robust assay from a large cohort of patients with tetanus. We have shown that catecholamine values were related to cardiovascular parameters of blood pressure and heart rate on the day of measurement. We have also shown that catecholamines measured on day 5 are predictors of subsequent clinically diagnosed ANSD.

Median catecholamine values were higher in those who experienced ANSD and MV compared to those with MV alone after day 5 of hospitalization (Additional file 1: Table S5), supporting the concept of ANSD being the most severe form of tetanus. A limitation of our study is that the number of deaths was too low to examine the relationship with in-hospital mortality. The population from which our patients were selected (i.e., the randomized controlled trial) meant that we did not include many of the most severe patients—i.e., those requiring MV early during hospitalization. In addition, we excluded those receiving vasopressors from our analysis. The randomized controlled trial found no evidence of clinical ANSD in those allocated to intrathecal antitoxin and our findings support this as we saw no difference in catecholamines with intrathecal antitoxin compared to sham procedure (Additional file 1: Table S6).

The non-linear relationship observed between catecholamines and cardiovascular parameters on day 5 is likely to reflect the highly complex and multi-level system involved in maintenance of cardiac output and end-organ perfusion, but may also show the disturbed physiology in tetanus. By choosing 24-h measurements, we aimed to reduce variability related to short plasma half-lives of catecholamines. As adrenaline is only released from the adrenal medulla, this method allows us to make reasonable estimations of plasma concentrations during the study period. However, noradrenaline is also released at sympathetic nerve endings, where neuronal reuptake ensures little enters the peripheral circulation. Consequently, urinary concentrations underestimate sympathetic activity and interpretation based exclusively on these will be limited. By examining only catecholamines, we have not accounted for the parasympathetic component of the autonomic nervous system. To do this, other approaches are required, for example heart rate variability analysis. Such understanding may enable better therapeutic advances in what remains one of the most challenging aspects of tetanus management.

## Conclusion

Urinary adrenaline and noradrenaline were associated with cardiovascular parameters and clinically relevant outcomes in tetanus. Data corroborate recently published evidence concerning clinical efficacy of intrathecal antitoxin in tetanus.

## Supplementary Information

**Additional file 1.** Additional tables and figures.

## Data Availability

All data generated or analyzed during this study are included in this published article [and its supplementary information files]. The dataset(s) supporting the conclusions of this article is (are) available in the Oxford University Research Archive (ORA).

## Abbreviations

ANSD: Autonomic nervous system dysfunction
APACHE II: Acute Physiology and Chronic Health Evaluation II
BMI: Body mass index
CI: Confidence interval
CMA: Causal mediation analysis
ELISA: Enzyme-linked immunosorbent assay
HR: Heart rate
HTD: Hospital for Tropical Diseases
ICU: Intensive care unit
IM: Intramuscular treatment
IT: Intrathecal treatment
MV: Mechanical ventilation
OR: Odds ratio
SD: Standard deviation
SOFA: Sequential Organ Failure Assessment
SBP: Systolic blood pressure
