# Supplementary material for: Urinary catecholamine excretion, cardiovascular variability, and outcomes in tetanus
Source: Trop Med Health. 2023 Mar 30;51:20. doi: 10.1186/s41182-023-00512-0 (PMC10061701; doi:10.1186/s41182-023-00512-0)
Supplement: Supplementary file 1 — Additional file 1. Additional tables and figures. [file 41182_2023_512_MOESM1_ESM.docx]

**Additional file 1**

**Entry and exclusion criteria for randomized controlled trial**

**Entry Criteria**

• All adult patients (≥16 years old) with a clinical diagnosis of generalized tetanus admitted to the intensive care unit (ICU) at the Hospital for Tropical Diseases, Ho Chi Minh City, Vietnam.

**Exclusion Criteria**

• For pilot phase only: Prior administration of antitoxin during this episode.

• Contra-indication to use of human or equine antitoxin or uncertainty about previous antitoxin treatment.

• Contra-indication to lumbar puncture.

• Already receiving mechanical ventilation or expected to require this before intrathecal injection can be given.

• Pregnancy.

• Informed consent not obtained.

**Figure: Study flow chart**

Adult patients with generalized tetanus enrolled in 2x2 factorial study

(n=272)

Patients receiving inotropes or vasopressors (n=6)

No urine sample obtained (n=3)

269 adults with Day 5 urine collections

(n=269)

Patients with samples for analysis

(n=263)

**Table S2.1.** Multivariate logistic regression models for associations between 24-hour urinary adrenaline (nmol/day) (on a log base 10 scale) and autonomic nervous system dysfunction (ANSD) after exclusion of patients developed ANSD before day 5 (n=240).

| **Characteristics** | **Summary** | | **Unadjusted** | | | **Adjusted 1^2^** | | | **Adjusted 2^2^** | | |
| --- | --- | --- | --- | --- | --- | --- | --- | --- | --- | --- | --- |
|  | **No ANSD,**  **N = 214 (89%)** | **ANSD,**  **N = 26 (11%)** | **OR^1^** | **95% CI^1^** | **P-value** | **OR^1^** | **95% CI^1^** | **P-value** | **OR^1^** | **95% CI^1^** | **P-value** |
| Log10 (Adrenaline (nmol/day)) | 2.18 [1.85, 2.50] | 2.75 [2.50, 2.92] | 15.4 | 5.3; 52.1 | <0.001 | 17.2 | 5.8; 60.7 | <0.001 | 8.9 | 2.4; 37.6 | 0.002 |
| Age | 48 [39, 60] | 46 [37, 50] |  |  |  | 0.98 | 0.94; 1.01 | 0.195 | 0.98 | 0.94; 1.02 | 0.344 |
| Sex |  |  |  |  |  |  |  |  |  |  |  |
| Female | 35 (16%) | 4 (15%) |  |  |  | - | - |  | - | - |  |
| Male | 179 (84%) | 22 (85%) |  |  |  | 0.41 | 0.11; 1.74 | 0.195 | 0.48 | 0.12; 2.21 | 0.322 |
| Intrathecal treatment |  |  |  |  |  |  |  |  |  |  |  |
| Sham procedure | 106 (50%) | 12 (46%) |  |  |  | - | - |  | - | - |  |
| Intrathecal treatment | 108 (50%) | 14 (54%) |  |  |  | 1.22 | 0.49; 3.05 | 0.669 | 0.87 | 0.32; 2.30 | 0.771 |
| Intramuscular treatment |  |  |  |  |  |  |  |  |  |  |  |
| Equine IM | 108 (50%) | 13 (50%) |  |  |  | - | - |  | - | - |  |
| Human IM | 106 (50%) | 13 (50%) |  |  |  | 0.79 | 0.32; 1.94 | 0.604 | 0.74 | 0.28; 1.89 | 0.527 |
| Total dose of benzodiazepines (100mg) | 22 [8, 27] | 30 [24, 32] |  |  |  |  |  |  | 1.00 | 0.94; 1.06 | 0.958 |
| Total dose of pipecuronium (100mg) | 0 [0, 0.52] | 1.31 [0.79, 1.92] |  |  |  |  |  |  | 2.93 | 1.58; 5.59 | <0.001 |
| ^1^ OR = Odds Ratio per one unit increased on a log base 10 scale of the urinary catecholamine excretion measured (nmol/day), CI = Confidence Interval  ^2^ **Adjusted 1:** Multivariate analyses adjusted for age, sex, intrathecal and intramuscular treatment interventions  ^2^ **Adjusted 2:** Multivariate analyses adjusted for age, sex, intrathecal and intramuscular treatment interventions, and total dose of benzodiazepines and pipecuronium during the first 5 days | | | | | | | | | | | |

**Table S2.2.** Multivariate logistic regression models for associations between 24-hour urinary noradrenaline (nmol/day) (on a log base 10 scale) and autonomic nervous system dysfunction (ANSD) after exclusion of patients developed ANSD before day 5 (n=240).

| **Characteristics** | **Summary** | | **Unadjusted** | | | **Adjusted 1^2^** | | | **Adjusted 2^2^** | | |
| --- | --- | --- | --- | --- | --- | --- | --- | --- | --- | --- | --- |
|  | **No ANSD,**  **N = 214 (89%)** | **ANSD,**  **N = 26 (11%)** | **OR^1^** | **95% CI^1^** | **P-value** | **OR^1^** | **95% CI^1^** | **P-value** | **OR^1^** | **95% CI^1^** | **P-value** |
| Log10 (Noradrenaline (nmol/day)) | 2.71 [2.45, 3.06] | 3.17 [2.98, 3.40] | 18.0 | 5.6; 69.3 | <0.001 | 26.6 | 7.7; 110.3 | <0.001 | 11.4 | 2.9; 52.5 | <0.001 |
| Age | 48 [39, 60] | 46 [37, 50] |  |  |  | 0.96 | 0.92; 0.00 | 0.016 | 0.97 | 0.93; 1.01 | 0.108 |
| Sex |  |  |  |  |  |  |  |  |  |  |  |
| Female | 35 (16%) | 4 (15%) |  |  |  | - | - |  | - | - |  |
| Male | 179 (84%) | 22 (85%) |  |  |  | 0.71 | 0.20; 2.92 | 0.607 | 0.68 | 0.18; 2.97 | 0.585 |
| Intrathecal treatment |  |  |  |  |  |  |  |  |  |  |  |
| Sham procedure | 106 (50%) | 12 (46%) |  |  |  | - | - |  | - | - |  |
| Intrathecal treatment | 108 (50%) | 14 (54%) |  |  |  | 1.27 | 0.51; 3.22 | 0.609 | 0.83 | 0.30; 2.24 | 0.713 |
| Intramuscular treatment |  |  |  |  |  |  |  |  |  |  |  |
| Equine IM | 108 (50%) | 13 (50%) |  |  |  | - | - |  | - | - |  |
| Human IM | 106 (50%) | 13 (50%) |  |  |  | 0.70 | 0.28; 1.74 | 0.440 | 0.65 | 0.24; 1.68 | 0.375 |
| Total dose of benzodiazepines (100mg) | 22 [8, 27] | 30 [24, 32] |  |  |  |  |  |  | 1.01 | 0.96; 1.07 | 0.626 |
| Total dose of pipecuronium (100mg) | 0 [0, 0.52] | 1.31 [0.79, 1.92] |  |  |  |  |  |  | 2.76 | 1.48; 5.34 | <0.001 |
| ^1^ OR = Odds Ratio per one unit increased on a log base 10 scale of the urinary catecholamine excretion measured (nmol/day), CI = Confidence Interval  ^2^ **Adjusted 1:** Multivariate analyses adjusted for age, sex, intrathecal and intramuscular treatment interventions  ^2^ **Adjusted 2:** Multivariate analyses adjusted for age, sex, intrathecal and intramuscular treatment interventions, and total dose of benzodiazepines and pipecuronium during the first 5 days | | | | | | | | | | | |


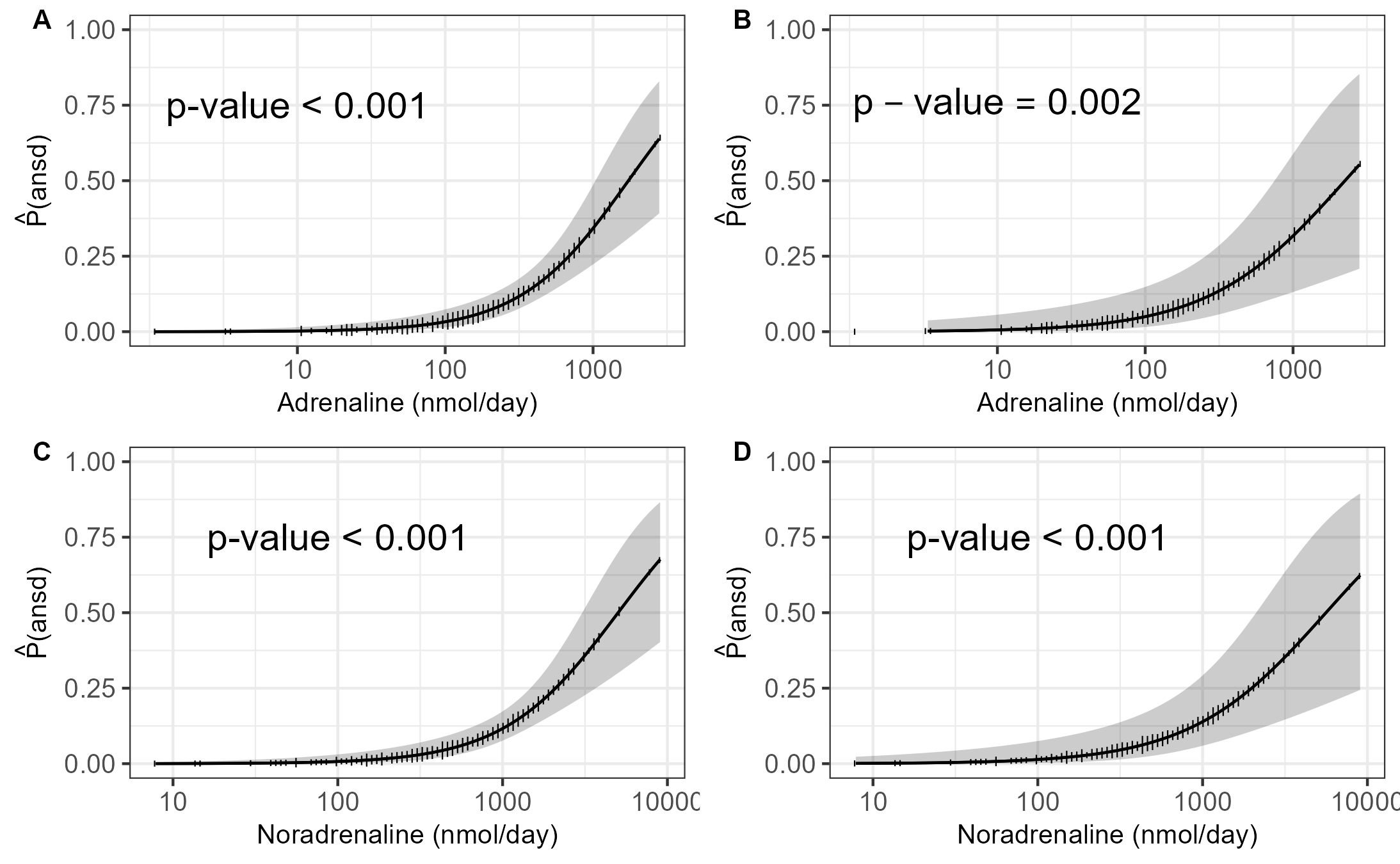


FIGURE S2.1. Models with linear trends of 24-hour urinary catecholamine excretion (nmol/day) and predicted probability of having autonomic nervous system dysfunction (ANSD) - (**A**) & (**C**): Unadjusted predictions, (**B**) & (**D**): All predictions are shown for age (48 years old), sex (male), intrathecal and intramuscular (equine intramuscular) treatment interventions, and total doses of benzodiazepines (2300 mg/day). The vertical lines represent the frequency counts of outcome variables. P-values indicate evidence for relationships between catecholamines and ANSD.


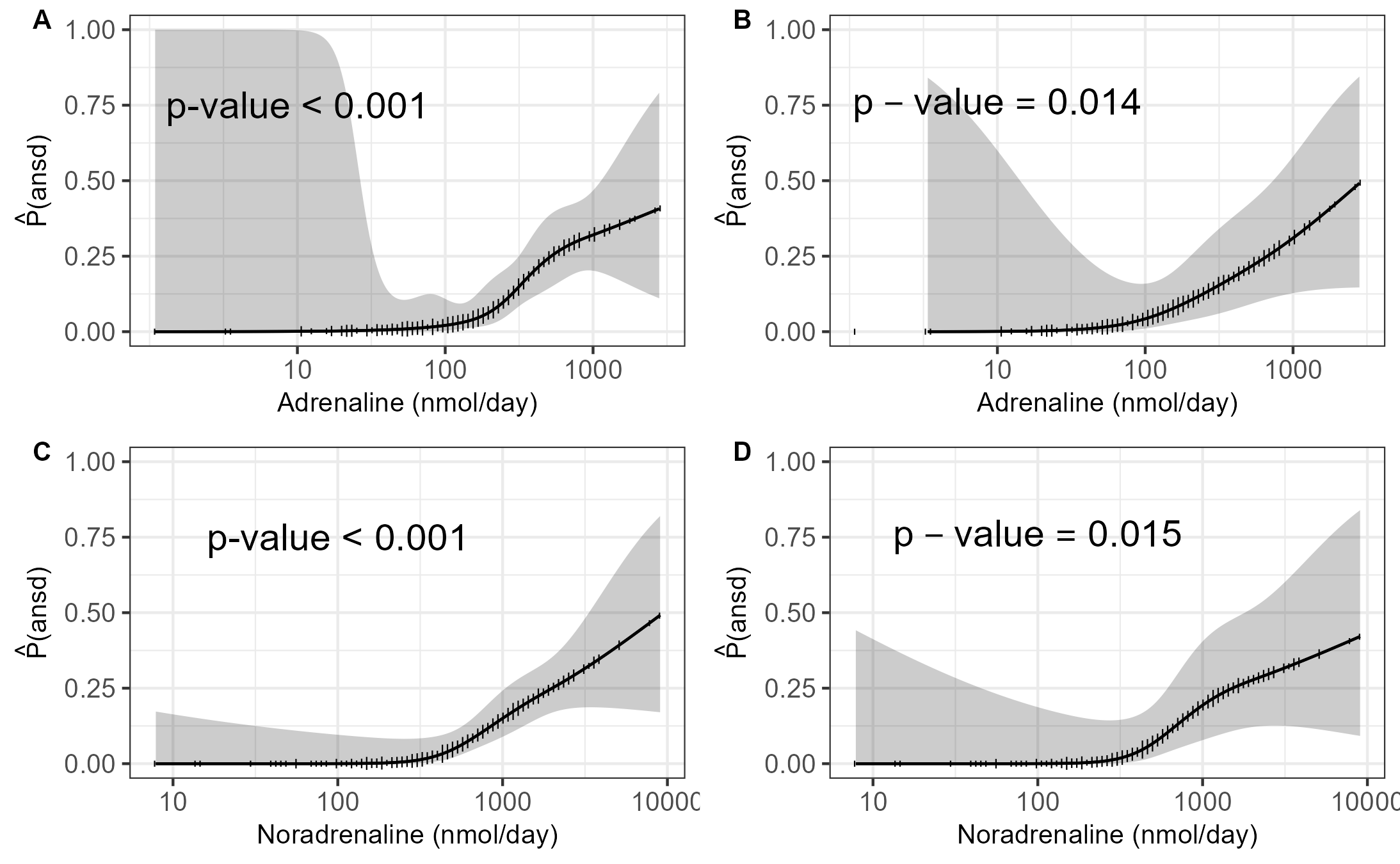


FIGURE S2.2. Models with restricted cubic splines of 24-hour urinary catecholamine excretion (nmol/day) and predicted probability of having autonomic nervous system dysfunction (ANSD) - (**A**) & (**C**): Unadjusted predictions, (**B**) & (**D**): All predictions are shown for age (48 years old), sex (male), intrathecal and intramuscular (equine intramuscular) treatment interventions, and total doses of benzodiazepines (2300 mg/day). The vertical lines represent the frequency counts of outcome variables. P-values indicate evidence for overall relationships between catecholamines and ANSD.

**Table S3.1.** Multivariate logistic regression models for associations between 24-hour urinary adrenaline (nmol/day) (on a log base 10 scale) and mechanical ventilation required (MV) after exclusion of patients required MV before day 5 (n =157).

| **Characteristics** | **Summary** | | **Unadjusted** | | | **Adjusted 1^2^** | | | **Adjusted 2^2^** | | |
| --- | --- | --- | --- | --- | --- | --- | --- | --- | --- | --- | --- |
|  | **No MV,**  **N = 140 (89%)** | **MV,**  **N = 17 (11%)** | **OR^1^** | **95% CI^1^** | **P-value** | **OR^1^** | **95% CI^1^** | **P-value** | **OR^1^** | **95% CI^1^** | **P-value** |
| Log10 (Adrenaline (nmol/day)) | 2.04 [1.76, 2.51] | 2.33 [2.12, 2.51] | 4.71 | 1.59, 15.24 | 0.007 | 5.75 | 1.62; 23.18 | 0.009 | 3.00 | 0.70; 14.09 | 0.149 |
| Age | 44 [38, 56] | 57 [42, 63] |  |  |  | 1.06 | 1.01; 1.11 | 0.013 | 1.07 | 1.02; 1.13 | 0.005 |
| Sex |  |  |  |  |  |  |  |  |  |  |  |
| Female | 20 (14%) | 4 (24%) |  |  |  | - | - |  | - | - |  |
| Male | 120 (86%) | 13 (76%) |  |  |  | 0.68 | 0.15; 3.42 | 0.622 | 0.66 | 0.15; 3.33 | 0.594 |
| Intrathecal treatment |  |  |  |  |  |  |  |  |  |  |  |
| Sham procedure | 66 (47%) | 12 (71%) |  |  |  | - | - |  | - | - |  |
| Intrathecal treatment | 74 (53%) | 5 (29%) |  |  |  | 0.48 | 0.14; 1.53 | 0.229 | 0.46 | 0.13, 1.47 | 0.202 |
| Intramuscular treatment |  |  |  |  |  |  |  |  |  |  |  |
| Equine IM | 74 (53%) | 8 (47%) |  |  |  | - | - |  | - | - |  |
| Human IM | 66 (47%) | 9 (53%) |  |  |  | 0.99 | 0.31; 3.11 | 0.983 | 1.07 | 0.33; 3.46 | 0.911 |
| Total dose of benzodiazepines (100mg) | 19 [6, 24] | 26 [16, 29] |  |  |  |  |  |  | 1.07 | 1.00; 1.16 | 0.077 |
| ^1^ OR = Odds Ratio per one unit increased on a log base 10 scale of the urinary catecholamine excretion measured (nmol/day), CI = Confidence Interval  ^2^ **Adjusted 1:** Multivariate analyses adjusted for age, sex, intrathecal and intramuscular treatment interventions  ^2^ **Adjusted 2:** Multivariate analyses adjusted for age, sex, intrathecal and intramuscular treatment interventions, and total dose of benzodiazepines during the first 5 days | | | | | | | | | | | |

**Table S3.2.** Multivariate logistic regression models for associations between 24-hour urinary noradrenaline (nmol/day) (on a log base 10 scale) and mechanical ventilation required (MV) after exclusion of patients required MV before day 5 (n =157).

| **Characteristics** | **Summary** | | **Unadjusted** | | | **Adjusted 1^2^** | | |  | **Adjusted 2^2^** |  |
| --- | --- | --- | --- | --- | --- | --- | --- | --- | --- | --- | --- |
|  | **No MV,**  **N = 140 (89%)** | **MV,**  **N = 17 (11%)** | **OR^1^** | **95% CI^1^** | **P-value** | **OR^1^** | **95% CI^1^** | **P-value** | **OR^1^** | **95% CI^1^** | **P-value** |
| Log10 (Noradrenaline (nmol/day)) | 2.59 [2.33, 2.84] | 3.02 [2.73, 3.14] | 10.77 | 2.89, 49.18 | <0.001 | 6.94 | 1.62; 35.82 | 0.013 | 4.23 | 0.94; 22.81 | 0.074 |
| Age | 44 [38, 56] | 57 [42, 63] |  |  |  | 1.04 | 0.99; 1.09 | 0.096 | 1.06 | 1.01; 1.12 | 0.026 |
| Sex |  |  |  |  |  |  |  |  |  |  |  |
| Female | 20 (14%) | 4 (24%) |  |  |  | - | - |  | - | - |  |
| Male | 120 (86%) | 13 (76%) |  |  |  | 0.86 | 0.21; 4.17 | 0.844 | 0.73 | 0.17; 3.59 | 0.680 |
| Intrathecal treatment |  |  |  |  |  |  |  |  |  |  |  |
| Sham procedure | 66 (47%) | 12 (71%) |  |  |  | - | - |  | - | - |  |
| Intrathecal treatment | 74 (53%) | 5 (29%) |  |  |  | 0.52 | 0.14; 1.67 | 0.283 | 0.48 | 0.13; 1.61 | 0.249 |
| Intramuscular treatment |  |  |  |  |  |  |  |  |  |  |  |
| Equine IM | 74 (53%) | 8 (47%) |  |  |  | - | - |  | - | - |  |
| Human IM | 66 (47%) | 9 (53%) |  |  |  | 0.99 | 0.31; 3.13 | 0.981 | 1.06 | 0.32; 3.47 | 0.927 |
| Total dose of benzodiazepines (100mg) | 19 [6, 24] | 26 [16, 29] |  |  |  |  |  |  | 1.08 | 1.01; 1.16 | 0.033 |
| ^1^ OR = Odds Ratio per one unit increased on a log base 10 scale of the urinary catecholamine excretion measured (nmol/day), CI = Confidence Interval  ^2^ **Adjusted 1:** Multivariate analyses adjusted for age, sex, intrathecal and intramuscular treatment interventions  ^2^ **Adjusted 2:** Multivariate analyses adjusted for age, sex, intrathecal and intramuscular treatment interventions, and total dose of benzodiazepines during the first 5 days | | | | | | | | | | | |


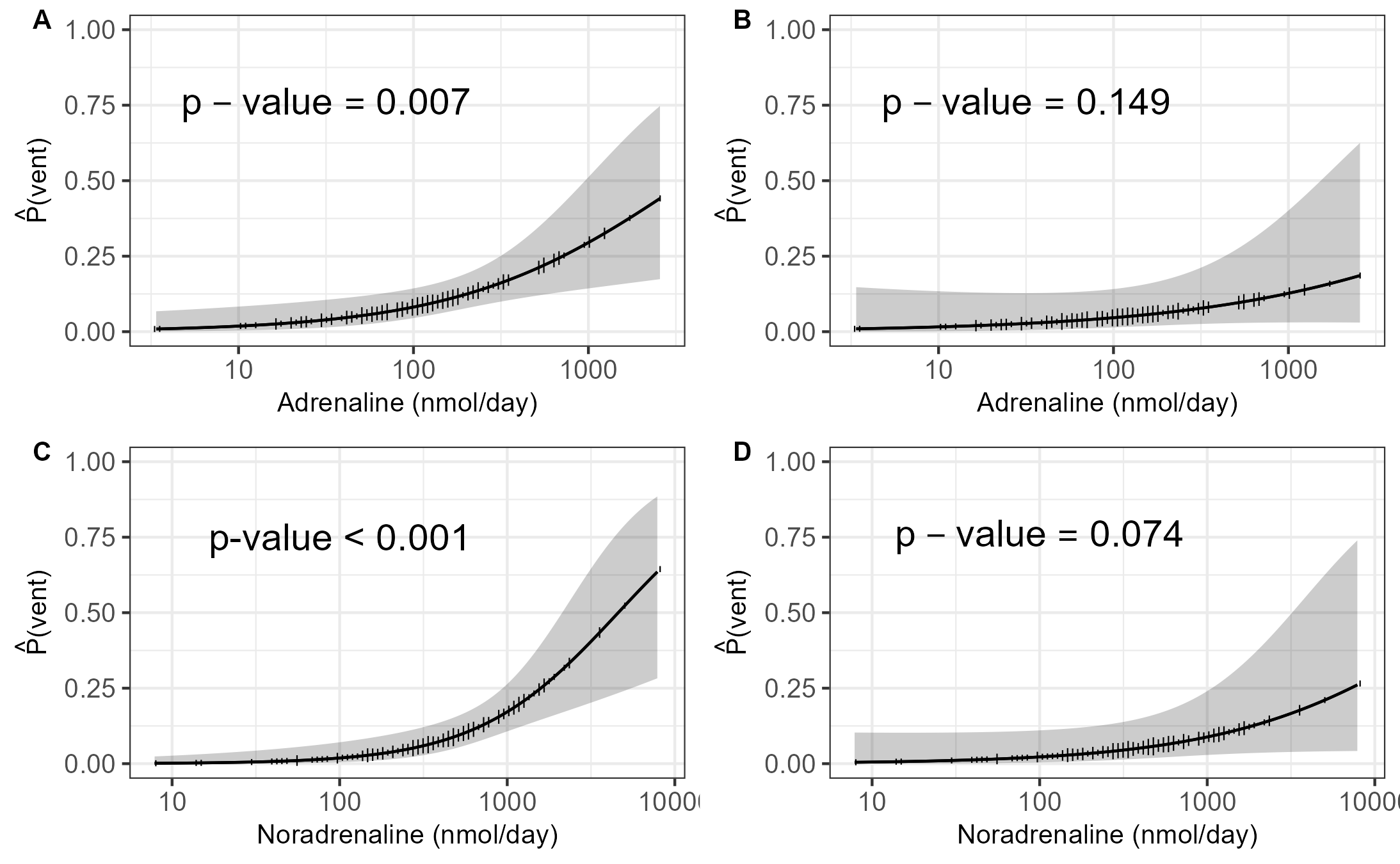


FIGURE S3.1. Models with linear trends of 24-hour urinary catecholamine excretion (nmol/day) and predicted probability of requiring mechanical ventilation (MV) - (**A**) & (**C**): Unadjusted predictions, (**B**) & (**D**): All predictions are shown for age (48 years old), sex (male), intrathecal and intramuscular (equine intramuscular) treatment interventions, and total doses of benzodiazepines (2300 mg/day). The vertical lines represent the frequency counts of outcome variables. P-values for overall relationships between catecholamine and MV.


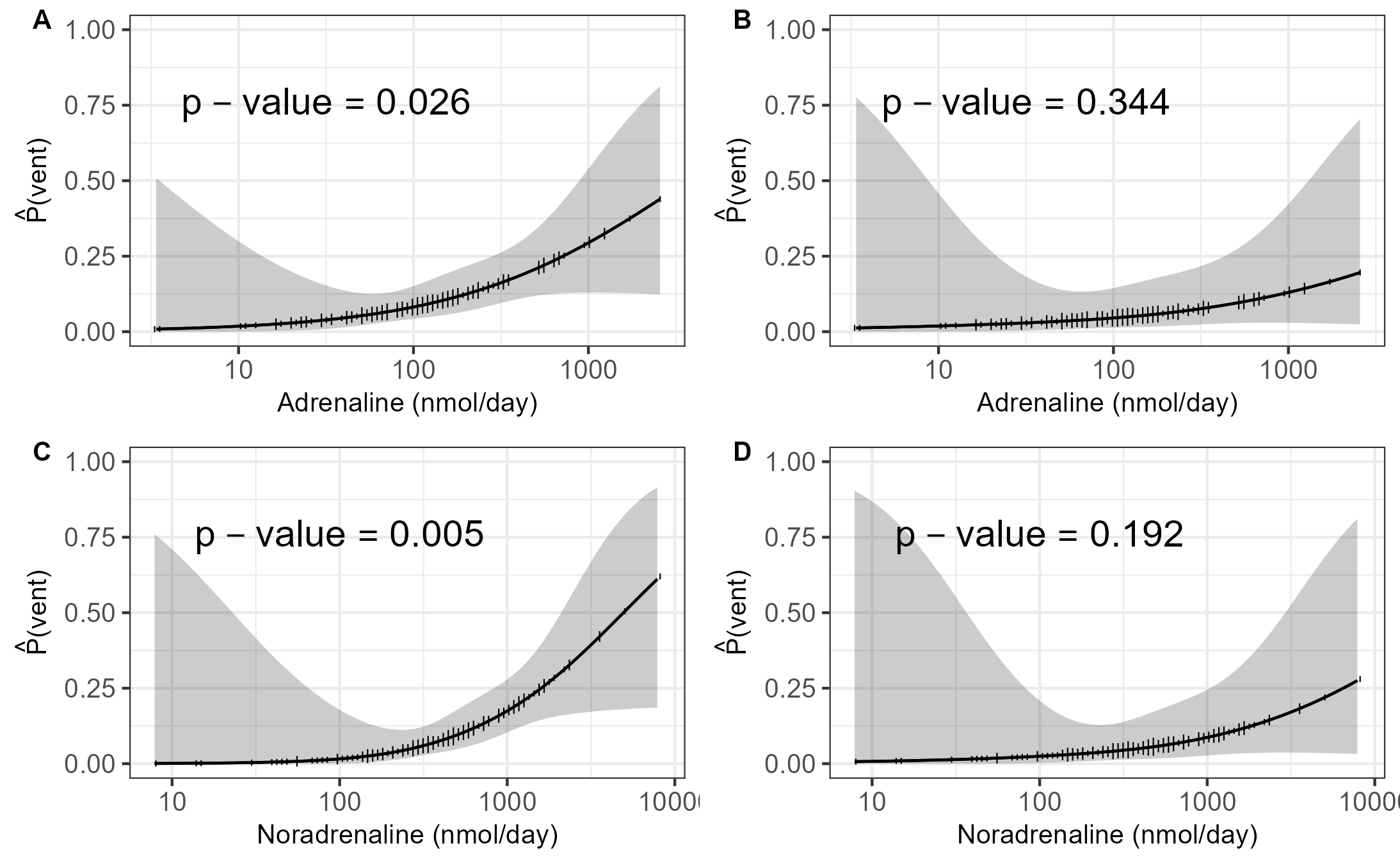


S FIGURE 3.2. Models with restricted cubic splines of 24-hour urinary catecholamine excretion (nmol/day) and predicted probability of requiring mechanical ventilation (MV) - (**A**) & (**C**): Unadjusted predictions, (**B**) & (**D**): All predictions are shown for age (48 years old), sex (male), intrathecal and intramuscular (equine intramuscular) treatment interventions, and total doses of benzodiazepines (2300 mg/day). The vertical lines represent the frequency counts of outcome variables. P-values for overall relationships between catecholamines and MV.

**Table S4.1**. Multivariate linear regression models for associations between 24-hour urinary adrenaline (nmol/day) (on a log base 10 scale) and length of ICU stay (days) (on a log base 10 scale).

| **Characteristics** | **Univariate** | | | **Adjusted 1^2^** | | | **Adjusted 2^2^** | | |
| --- | --- | --- | --- | --- | --- | --- | --- | --- | --- |
|  | **Beta^1^** | **95% CI^1^** | **P-value** | **Beta^1^** | **95% CI^1^** | **P-value** | **Beta^1^** | **95% CI^1^** | **P-value** |
| Log10 (Adrenaline (nmol/day)) | 0.23 | 0.18; 0.29 | <0.001 | 0.23 | 0.18; 0.29 | <0.001 | 0.07 | 0.02; 0.12 | 0.005 |
| Age |  |  |  | 0.004 | 0.002; 0.006 | 0.001 | 0.005 | 0.004; 0.007 | <0.001 |
| Sex |  |  |  |  |  |  |  |  |  |
| Female |  |  |  | - | - |  | - | - |  |
| Male |  |  |  | -0.02 | -0.11; 0.07 | 0.638 | -0.03 | -0.10; 0.04 | 0.343 |
| Intrathecal treatment |  |  |  |  |  |  |  |  |  |
| Sham procedure |  |  |  | - | - |  | - | - |  |
| Intrathecal treatment |  |  |  | 0.01 | -0.06; 0.06 | 0.967 | -0.04 | -0.09; 0.01 | 0.062 |
| Intramuscular treatment |  |  |  |  |  |  |  |  |  |
| Equine IM |  |  |  | - | - |  | - | - |  |
| Human IM |  |  |  | -0.01 | -0.06; 0.06 | 0.932 | -0.02 | -0.07; 0.02 | 0.306 |
| Total dose of benzodiazepines (100mg) |  |  |  |  |  |  | 0.009 | 0.007; 0.012 | <0.001 |
| Total dose of pipecuronium (100mg) |  |  |  |  |  |  | 0.16 | 0.13; 0.20 | <0.001 |
| ^1^ Beta = Increase in length of ICU stay on a log base 10 scale with one unit increase of the urinary catecholamine excretion measured (nmol/day) on a log base 10 scale, CI = Confidence Interval  ^2^ **Adjusted 1:** Multivariate analyses: adjusted for age, sex, intrathecal and intramuscular treatment interventions  ^2^ **Adjusted 2:** Multivariate analyses: adjusted for age, sex, intrathecal and intramuscular treatment interventions, and total dose of medications (benzodiazepines, pipecuronium) during the first 5 days | | | | | | | | | |

**Table S4.2.** Multivariate linear regression models for associations between 24-hour urinary noradrenaline (nmol/day) (on a log base 10 scale) and length of ICU stay (days) (on a log base 10 scale).

| **Characteristics** | **Univariate** | | | **Adjusted 1^2^** | | | **Adjusted 2^2^** | | |
| --- | --- | --- | --- | --- | --- | --- | --- | --- | --- |
|  | **Beta^1^** | **95% CI^1^** | **P-value** | **Beta^1^** | **95% CI^1^** | **P-value** | **Beta^1^** | **95% CI^1^** | **P-value** |
| Log10 (Noradrenaline (nmol/day)) | 0.31 | 0.25; 0.36 | <0.001 | 0.30 | 0.24; 0.36 | <0.001 | 0.10 | 0.04; 0.15 | <0.001 |
| Age |  |  |  | 0.001 | -0.001; 0.004 | 0.208 | 0.004 | 0.003; 0.006 | <0.001 |
| Sex |  |  |  |  |  |  |  |  |  |
| Female |  |  |  | - | - |  | - | - |  |
| Male |  |  |  | 0.01 | -0.08; 0.09 | 0.904 | -0.02 | -0.09; 0.04 | 0.479 |
| Intrathecal treatment |  |  |  |  |  |  |  |  |  |
| Sham procedure |  |  |  | - | - |  | - | - |  |
| Intrathecal treatment |  |  |  | 0.01 | -0.05; 0.06 | 0.867 | -0.04 | -0.09; 0.01 | 0.085 |
| Intramuscular treatment |  |  |  |  |  |  |  |  |  |
| Equine IM |  |  |  | - | - |  | - | - |  |
| Human IM |  |  |  | -0.02 | -0.08; 0.03 | 0.444 | -0.03 | -0.07; 0.02 | 0.203 |
| Total dose of benzodiazepines (100mg) |  |  |  |  |  |  | 0.009 | 0.007; 0.012 | <0.001 |
| Total dose of pipecuronium (100mg) |  |  |  |  |  |  | 0.15 | 0.11; 0.19 | <0.001 |
| ^1^ Beta = Increase in length of ICU stay on a log base 10 scale with one unit increase of the urinary catecholamine excretion measured (nmol/day) on a log base 10 scale, CI = Confidence Interval  ^2^ **Adjusted 1:** Multivariate analyses: adjusted for age, sex, intrathecal and intramuscular treatment interventions  ^2^ **Adjusted 2:** Multivariate analyses: adjusted for age, sex, intrathecal and intramuscular treatment interventions, and total dose of medications (benzodiazepines, pipecuronium) during the first 5 days | | | | | | | | | |


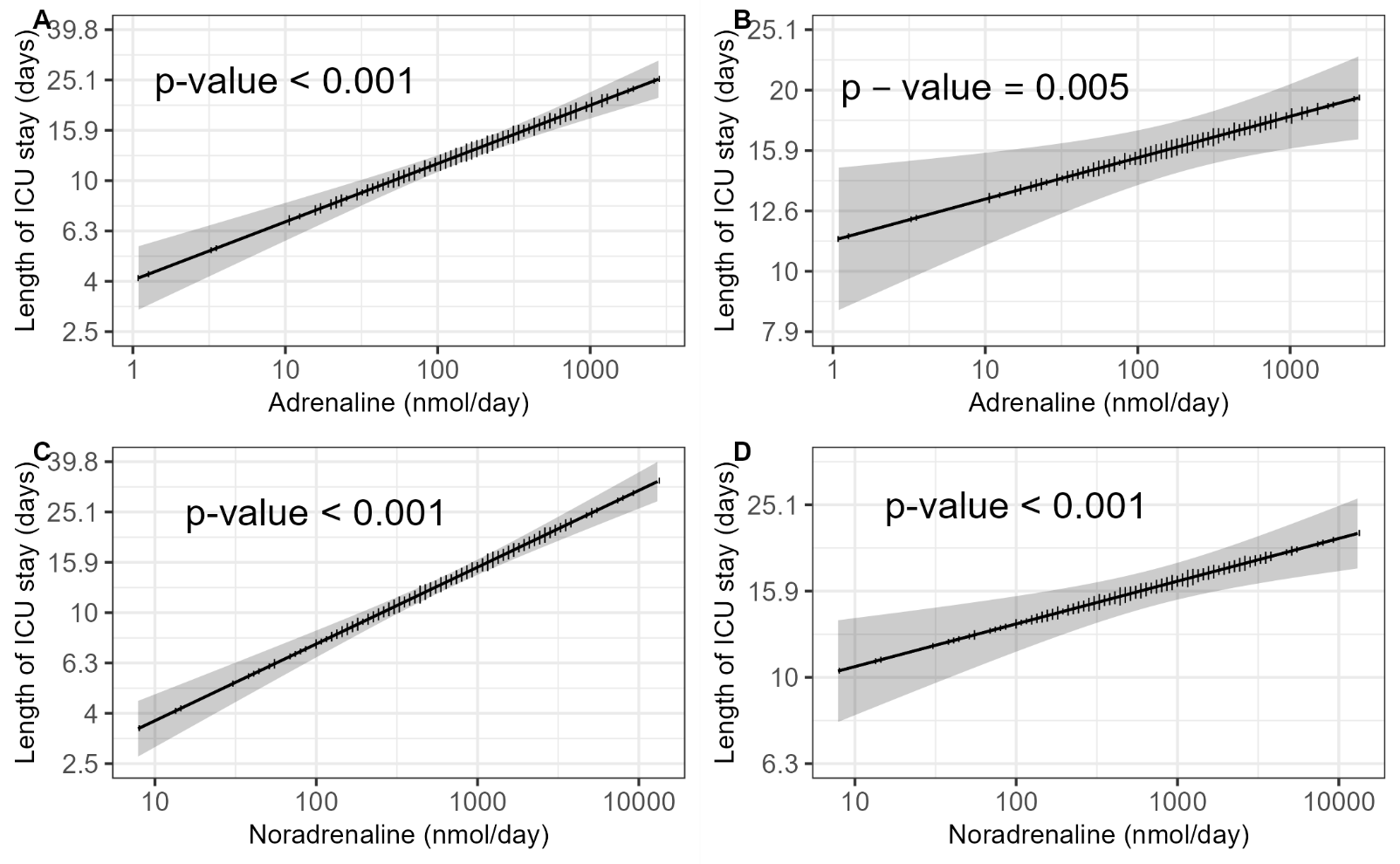


FIGURE S4.1. Models with linear trends of 24-hour urinary catecholamine excretion (nmol/day) and length of ICU stay (days). - (**A**) & (**C**): Unadjusted predictions, (**B**) & (**D**): All predictions are shown for age (48 years old), sex (male), intrathecal and intramuscular (equine intramuscular) treatment interventions, and total doses of benzodiazepines (2300 mg/day). The vertical lines represent the frequency counts of outcome variables. P-values indicate evidence for relationships between catecholamine and length of ICU stay (days).


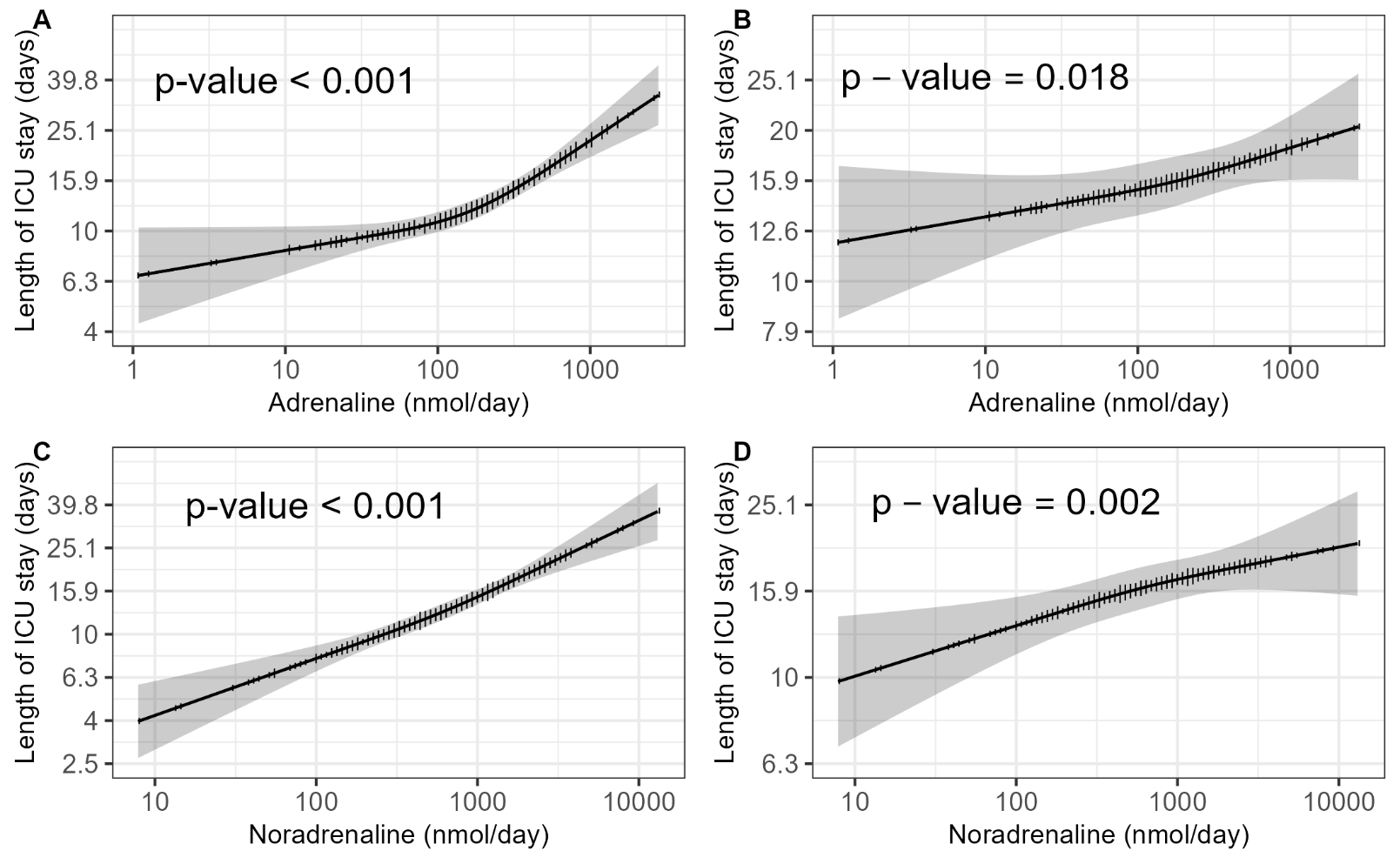


FIGURE S4.2. Models with restricted cubic splines of 24-hour urinary catecholamine excretion (nmol/day) and length of ICU stay (days). - (A) & (C): Unadjusted predictions, (B) & (D): All predictions are shown for age (48 years old), sex (male), intrathecal and intramuscular (equine intramuscular) treatment interventions, and total doses of benzodiazepines (2300 mg/day). The vertical lines represent the frequency counts of outcome variables. P-values indicate evidence for overall relationships between catecholamine and length of ICU stay (days).

**Table S5.** 24-hour urinary catecholamine excretion on the 5th day of treatment among no mechanical ventilation (MV) required, MV only, and MV required and developed autonomic nervous system dysfunction (ANSD) groups after exclusion of patients required MV or ANSD before day 5 (n =157).

| **Catecholamines** | **Median [Q1, Q3]** | | |  |
| --- | --- | --- | --- | --- |
|  | **No MV,**  **N = 140 (89%)** | **MV only,**  **N = 12 (8%)** | **MV and ANSD,**  **N = 5 (3%)** | **P-value** |
| Log10 (Adrenaline (nmol/day)) | 2.04 [1.76, 2.28] | 2.18 [2.03, 2.49] | 2.51 [2.29, 3.00] | 0.030 |
| Log10 (Noradrenaline (nmol/day)) | 2.59 [2.33, 2.84] | 3.05 [2.66, 3.10] | 2.99 [2.80, 3.22] | 0.002 |

P-value indicates evidence from Kruskal-Wallis rank sum test for a difference between groups

**Table S6.** 24-hour urinary catecholamine excretion on the 5th day of treatment versus intrathecal and intramuscular treatment

| **Catecholamines** | **Median [Q1, Q3]** | |  | **Median [Q1, Q3]** | |  |
| --- | --- | --- | --- | --- | --- | --- |
|  | **Sham procedure,**  **N = 129 (49%)** | **Intrathecal treatment,**  **N = 134 (51%)** | **P-value** | **Equine IM,**  **N = 132 (50%)** | **Human IM,**  **N = 131 (50%)** | **P-value** |
| Log10 (Adrenaline (nmol/day)) | 2.25 [1.99, 2.62] | 2.24 [1.86, 2.65] | 0.496 | 2.20 [1.84, 2.52] | 2.33 [2.02, 2.68] | 0.061 |
| Log10 (Noradrenaline (nmol/day)) | 2.86 [2.54, 3.18] | 2.79 [2.48, 3.13] | 0.348 | 2.70 [2.46, 3.11] | 2.91 [2.64, 3.19] | 0.063 |

P-value indicates evidence from Wilcoxon rank sum test for a difference between groups
